# Uterus transplantation — indications, technique, and results

**DOI:** 10.1590/0102-67202025000054e1923

**Published:** 2026-02-13

**Authors:** Wellington ANDRAUS, Dani EJSENBERG, Daniel Reis WAISBERG, Alexandre Chagas SANTANA, Liliana DUCATTI, Rubens Macedo ARANTES, Rodrigo Bronze de MARTINO, Vinicius Rocha SANTOS, Rafael Soares PINHEIRO, Luciana LEIS, Maciana Santos SILVA, Luciana Bertocco HADDAD, José Maria SOARES, Pedro Augusto Araujo MONTELEONE, Edmund Chada BARACAT

**Affiliations:** 1 Universidade de São Paulo, Faculty of Medicine, Department of Gastroenterology – São Paulo (SP), Brazil.

**Keywords:** Organ Transplantation, Tissue Donors, Infertility, Uterus, Hysterectomy, Transplante de órgãos, Doadores de Tecidos, Infertilidade, Utero, Histerectomia

## Abstract

Uterus transplantation was a transformative innovation in reproductive medicine and organ transplantation in general, and an alternative for the treatment of infertility. The problem of infertility affects 8–12% of the population of reproductive age, causing an enormous social impact. Uterus transplantation, a relatively new treatment, has emerged as an excellent option for couples with absolute uterine infertility. The first uterus transplant performed was in 2000, in Saudi Arabia. At this same time, a Swedish researcher began several experimental works with uterine transplantation in different animal models. Only more than a decade after the first attempt in humans was a second case performed, in Turkey, in 2011. The first transplant in the Americas was performed in the United States of America, in 2016, with a deceased donor. In the same year, in Brazil, the group from Hospital das Clínicas, Faculty of Medicine, University of São Paulo, performed the first uterus transplant in Latin America, also with a deceased donor. This Brazilian case resulted in the world’s first birth from a deceased donor uterus transplant in December 2017, making Brazil and Hospital das Clínicas in a vanguard position in the world transplant scenario. Even so, we have today more than 100 transplants performed on the planet, with the birth of more than 70 children.

## ARTICLE HIGHLIGHTS

Uterus transplantation was a transformative innovation in reproductive medicine and organ transplantation.

It is an alternative for the treatment of absolute uterine factor infertility.

Mayer-Rokitansky-Küster-Hauser syndrome is the main cause of uterine agenesis.

This Brazilian case resulted in the world’s first birth from a deceased donor uterus transplant in December 2017.

Today, more than 100 transplants are performed on the planet, with the birth of more than 70 children.

## Summary

**CENTRAL MESSAGE:**

Uterus transplantation has undoubtedly been a transformative innovation in reproductive medicine and organ transplantation. It comes as an alternative for treating infertility of an absolute uterine cause. Among these causes, we have women who were born without this organ, or there was a need to perform a hysterectomy, or they have a non-functioning uterus, such as severe malformations (severe bicornuate uterus, Asherman syndrome). Modern society has increasingly respected each individual’s decision, provided they are informed of all alternatives and risks involved. In this way, uterine transplantation has become an important alternative for patients with infertility due to uterine causes.

**PERSPECTIVES:**

Uterus transplantation is a new modality among organ transplants. It has undoubtedly brought great innovation and excellent results to the therapeutic armamentarium for infertility of uterine cause. Uterus transplantation is a great example where multidisciplinarity, in which each one contributes with their expertise, enables the realization of a greater feat. The gain of experience, technology, and minimally invasive surgeries will certainly bring even better results.

## VISUAL ABSTRACT

**Figure:**
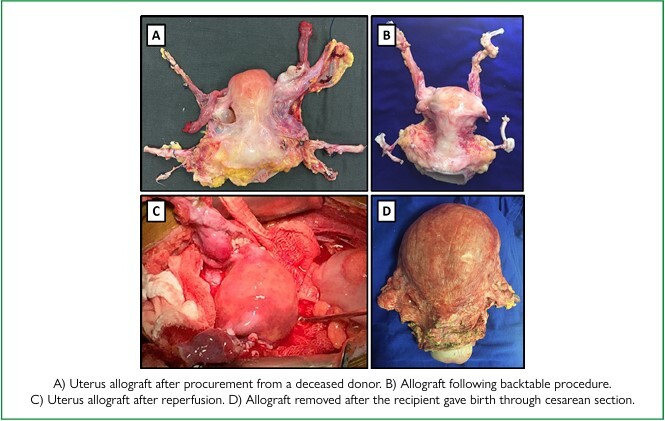

## RESUMO

O transplante de útero foi uma inovação transformadora na medicina reprodutiva e no transplante de órgãos em geral, além de ser uma alternativa para o tratamento da infertilidade. O problema da infertilidade afeta de 8 a 12% da população em idade reprodutiva, causando enorme impacto social. O transplante de útero, um tratamento relativamente novo, surgiu como uma excelente opção para casais com infertilidade uterina absoluta. O primeiro transplante de útero realizado foi em 2000, na Arábia Saudita. Nessa mesma época, um grupo na Suécia iniciou diversos trabalhos experimentais, com transplante uterino, em diferentes modelos animais. Apenas mais de uma década após a primeira tentativa em humanos, um segundo caso foi realizado, na Turquia, em 2011. O primeiro transplante nas Américas foi realizado nos Estados Unidos, em 2016, com doadora falecida. No mesmo ano, no Brasil, o grupo do Hospital das Clínicas da Faculdade de Medicina da Universidade de São Paulo (HCFMUSP) realizou o primeiro transplante de útero na América Latina, também com doadora falecida. Este caso brasileiro resultou no primeiro nascimento no mundo, a partir de um transplante de útero de doadora falecida, em dezembro de 2017, colocando o Brasil e o Hospital das Clínicas na vanguarda do cenário mundial de transplantes. Atualmente, temos hoje mais de 100 transplantes realizados no planeta, com o nascimento de mais de 70 crianças.

## INTRODUCTION

 Uterus transplantation was a transformative innovation in reproductive medicine and organ transplantation in general^10,21,25,27^. It is an alternative for the treatment of absolute uterine factor infertility^18^. Studies show that approximately 90% of young women wish to have children, and the World Health Organization defines infertility as: "the inability of a couple to achieve conception or carry a conception to term after 12 months or more of regular, unprotected sexual intercourse"^35,37^. 

 The problem of infertility affects 8–12% of the population of reproductive age, causing an enormous social impact^34^. Additionally, couples with infertility present more anxiety problems, decreased work productivity, and lower quality of life indices. The pursuit of fertility is supported by the Universal Declaration of Human Rights of 1948, which states: "Men and women of full age, without any limitation due to race, nationality, or religion, have the right to marry and to found a family"^33^. 

 Among all causes, infertility of exclusively uterine origin accounts for 3–5% of cases. It is estimated that there are about 150,000 women in Europe with this condition. Surrogate uterus and adoption are options in these cases for these couples to have children^27^. However, the surrogate uterus alternative is prohibited in several countries, such as Japan and Sweden^7,19^. In Brazil, it is permitted, but regulated by the Federal Council of Medicine resolution, being allowed only for female relatives of one of the partners up to the fourth degree of kinship or non-relatives with approval from regional medical councils, in good health conditions, and no profit or commercial character may be involved. Furthermore, the temporary uterus donor is exposed to all the risks of pregnancy and childbirth^15^. 

 Modern society has increasingly respected each individual’s decision, after all the alternatives and risks involved have been clarified. 

 Uterus transplantation, a relatively new treatment, has emerged as an excellent option for couples with absolute uterine infertility. 

### History

 The first experimental studies in uterus transplantation date back to the 1960s, with the first reports being an attempt to solve problems with the fallopian tubes. In 1978, in vitro fertilization began, which revolutionized infertility treatment from then on and reduced interest in uterine transplantation^23,28,40^. 

 The first uterus transplant performed was in April 2000, in Saudi Arabia. Fageeh et al., in Jeddah, performed a transplant with a living donor, a 46-year-old, and implanted it in a 26-year-old recipient. This transplant was not successful, and the uterus was removed a few months after surgery due to vascular thrombosis and prolapse^14,28^. 

 At this same time, the Swedish group led by Dr. Mats Brännström began several experimental works with uterine transplantation in different animal models, from mice to sheep to baboons, in order to acquire experience in this completely new procedure^3,8,26^. 

 Only more than a decade after the first attempt in humans was a second case performed, this time in Antalya, Turkey. In 2011, Ozkan et al. performed a uterine transplant, this time with a 22-year-old deceased donor due to traumatic brain injury and a 21-year-old recipient with absence of uterus due to Mayer-Rokitansky-Küster-Hauser (MRKH) syndrome. In this case, the uterus remained viable; however, they did not achieve pregnancy after embryo transfers in subsequent years^24^. 

 The aforementioned Swedish group started its clinical program in 2012 and transplanted 9 women, all with living donors^6^. The world’s first birth from uterine transplantation occurred in 2014, from this series at Sahlgrenska Hospital, University of Gothenburg. This pioneering case involved the transplant from a 61-year-old donor to a 31-year-old recipient with MRKH syndrome. The recipient developed pre-eclampsia during pregnancy, and the baby was born premature at 31 weeks and 5 days, but both had good outcomes. In 2024, this Swedish boy gave a speech at the World Congress of Uterus Transplantation in Gothenburg on his 10th birthday^5^. 

 The first transplant in the Americas was performed by Kisu et al. in February 2016, in Cleveland, with a deceased donor. The uterus needed to be removed a few days after surgery due to a fungal infection^20^. In the same year, in September, in Brazil, the group from Hospital das Clínicas, Faculty of Medicine, University of São Paulo (HCFMUSP), performed the first uterus transplant in Latin America, also with a deceased donor^13,30^. 

 This Brazilian case resulted in the world’s first birth from a deceased donor uterus transplant in December 2017, marking Brazil’s and Hospital das Clínicas’ vanguard position in the world transplant scenario. This pioneering work had enormous worldwide repercussions after its publication in The Lancet in 2018^13^, registering the name of HCFMUSP in newspapers and news programs worldwide. 

 So much so that the website Altmetric, which ranks scientific publications according to their impact across all media, ranked it first in the Lancet journal that year, 17th in the same journal all-time, and among the top 5% of all articles ever ranked. Furthermore, it was the only University of São Paulo article included among the "top" 100 articles in the year it was published. 

### Indications

 The indications for transplantation are absolute uterine infertility causes^14,21,30^. Among these causes are women who were born without this organ, or there was a need to perform a hysterectomy, or they have a non-functioning uterus, such as severe malformations or adhesions (rudimentary uterus, Asherman’s syndrome)^14,21^. The estimated rate of infertility due to uterine causes is one in every 500 women^21,32^. 

 MRKH syndrome is the main cause of uterine agenesis. It occurs in one in every 4,500 women^14,32^. Although rare, if we consider the population of São Paulo of women of reproductive age, which is estimated at 12.2 million according to the Brazilian Institute of Geography and Statistics, we can see that we have a large number of patients with this condition who could opt for this therapeutic alternative^9^. 

 Hysterectomy is a surgery performed very frequently for different indications^38^. In the United States, about 500,000 hysterectomies are performed per year, with 40% of them in patients under 44 years of age^11^. Examples of causes leading to hysterectomy include hemorrhages during pregnancy or childbirth, leiomyomas, adenomyosis, and gynecological cancer, predominantly cervical^38^. 

 Postpartum bleeding is an important cause of maternal death^36^. The incidence varies from 0.13 to 5.38 cases per 1,000 childbirths^1,17,22^. It seems like a small percentage, but when we see the absolute number of deliveries only in São Paulo, which is around 500,000 childbirths per year, we better understand the dimension of the problem^31^. Furthermore, according todata from the National Cancer Institute, cervical cancer is responsible for more than 17,000 new cancer cases per year, with 44% being non-invasive^14,21,31^. 

 Adding to malignant tumors, we have benign tumors such as leiomyomas and adenomyosis, which are responsible for 5% of hysterectomies for benign diseases in women of reproductive age. Reinforcing the importance of hysterectomy as a promoter of infertility, in the United States, 50% of women candidates for surrogate uterus treatment had undergone this procedure^10^. 

### Technique

 The uterus transplant technique can be divided into donor and recipient surgery. There is the modality of living and deceased donors, so for didactic purposes, we will divide it into three procedures: living donor surgery, deceased donor surgery, and recipient surgery. 

### Living donor surgery

 The incision performed is infraumbilical midline. The uterus is dissected and freed from the peritoneum and ligaments. A careful and meticulous dissection of the ureter is performed, and to facilitate this step, catheters are placed inside it (double J). This dissection is very important because the ureter has intimate contact with the uterine vessels in its deepest path. 

 The uterine artery is dissected to its origin in the internal iliac artery bilaterally. The uterine vein, which sometimes forms a small plexus, is dissected to its entry into the internal iliac vein bilaterally. The utero/ovarian veins are also dissected. When all vessels are freed and fully dissected, we proceed to section the vagina near the uterine cervix (about 1.5–2 cm). 

 The vessels are then clamped; the uterine artery is removed with a segment of the internal iliac artery and the uterine vein with a "patch" of the internal iliac vein, and the utero/ovarian veins are sectioned near the ovaries. The graft is then removed, all vessels are ligated or sutured, and we proceed with closure of the abdominal wall. The double J catheters remain for at least 1 week after surgery^5,6^. 

### Deceased donor surgery

 The procedure occurs in conjunction with multi-organ procurement. The donor undergoes a xipho-pubic incision. The uterus is dissected and freed from the peritoneum and ligaments. We dissect the common, internal, and external iliac vessels bilaterally. We dissect the ureters and gonadal vessels. The internal iliac vessels are dissected and prepared. 

 After ligation of the aorta, near the bifurcation of the iliac arteries, the external iliac arteries are clamped distally, near the femoral arteries. Perfusion is then performed with preservation solution; normally, Custodiol (HTK) is used because it is less viscous, through cannulation of the common iliac arteries. Ice is placed in the pelvis, and at this moment, the cold ischemia time of the graft begins. After washing with preservation solution, the uterus is removed with the internal iliac vessels and ovarian vessels. The vagina is sectioned about 2 cm from the uterine cervix^2,13,24^. 

### "Back table" surgery

 The graft is kept cool in an ice-cold solution. It is perfused with preservation solution, normally Custodiol (HTK). The vessels are then carefully dissected and prepared for anastomosis during organ implantation in the recipient. Both uterine arteries are always implanted, performing the anastomosis of the internal iliac artery stump of the graft with the external iliac artery of the recipient. 

 The veins are evaluated at this stage, and if possible, all four veins are implanted. However, in some cases, one or two of the veins are extremely thin, less than 1 mm, or totally obstructed and are not capable of being implanted. The graft is then taken for implantation in the recipient^5,6^. 

### Recipient surgery

 An infraumbilical midline incision is performed. The vessels used for implantation are the external iliac vessels, which greatly facilitate dissection and implantation as they are more superficial. The vaginal vault is prepared but not opened. Venous and arterial anastomoses are then performed bilaterally, most often numbering six anastomoses, in the external iliac vessels (arteries and veins), with 7-0 Prolene suture, with continuous suture. 

**Figure 1:**
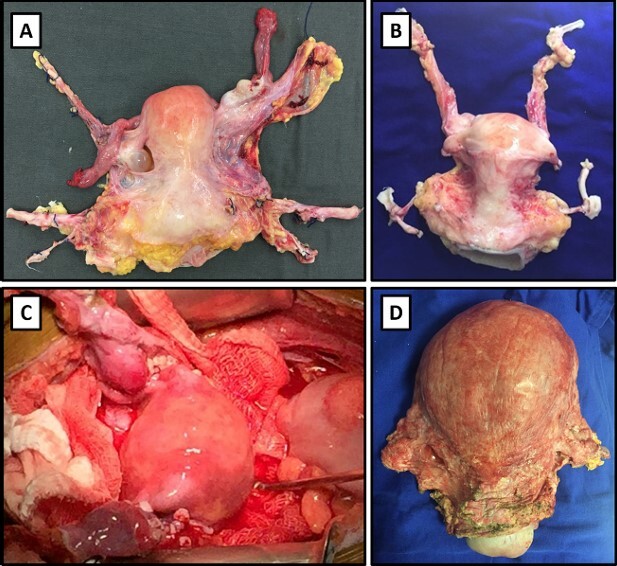
A) Uterus allograft after procurement from a deceased donor. B) Allograft following backtable procedure. C) Uterus allograft after reperfusion. D) Allograft removed after the recipient gave birth through cesarean section.

 At the completion of bilateral anastomoses, the uterus is revascularized. Hemostasis is performed. The recipient’s vaginal vault is then opened, and the vaginal stump of the graft is anastomosed with the recipient’s vaginal vault with separate Vicryl stitches. Care must be taken to avoid stenosis of this anastomosis, which can make evaluation of the graft in the postoperative period difficult through speculum examination and cervical biopsy, as well as embryo transfer^5,6^. The sequential surgical stages of the uterus allograft procedure are shown in Figure 1A–D. 

## RESULTS

 Despite being a relatively new procedure, with the first birth in 2014, having completed 10 years this year^5,12^, we have had a great expansion of transplant centers worldwide. The procedure, until recently, was experimental and performed only as research. Even so, we have today more than 100 transplants performed on the planet, with the birth of more than 70 children^32^. 

 The centers with the greatest worldwide experience are the University of Gothenburg in Sweden and Baylor University in Dallas, USA, each having performed about 20 cases of uterus transplantation^21,32^. 

 Rejection is not frequent, occurring in about 15% of patients, and can be reversed with medications in most cases. Other complications inherent to transplants may be present, such as vascular thrombosis, infections, and adverse effects of immunosuppression^16^. On the other hand, because the uterus is not a vital organ, although some cases have lost the graft, its removal did not cause any serious health problems to the recipient^12^. 

 The immunosuppression used in uterus transplantation is, comparatively with other transplanted solid organs, relatively light. The consensus of the International Society for Uterus Transplantation (ISUTx) is that it is acceptable to remain with the graft (uterus) for up to 5 years, which in most cases would allow two pregnancies, and this period of light immunosuppression brings a very small risk to recipients^12,16^. 

 The HCFMUSP experience is of three cases, the first two from deceased donors and the last from a living donor. The first was successful on the first embryo transfer only 7 months after transplantation. This allowed the patient to become pregnant, have her daughter, and remain only 18 months with the transplanted uterus, that is, only 18 months of immunosuppression. 

 The second case, performed in 2017, presented vascular thrombosis and required graft removal 2 days after transplantation. The patient had no complications from the surgery^2,13^. 

 After a temporary interruption of the program due to the pandemic, we resumed the program and performed in August 2024 a uterus transplant with a living donor. It is the first successful living donor uterus transplant case in Latin America. The patient is evolving very well and has already presented menstrual cycles. The plan is embryo transfer 4–6 months after transplantation. The donor, her sister, had no complications. Both remained only 6 days hospitalized after surgery. 

 The birth of a baby after uterus transplantation, which is considered the actual success of the procedure, occurs in 7080% of cases, which is an excellent rate. A recent review article on the topic in the European Journal of Transplantation 2024 confirms the good results of uterus transplantation in centers around the world and the increasing growth in the number of procedures performed^16^. 

### Perspectives

 The use of the robotic platform for living donor surgery is not exactly a perspective because it has already been performed by some centers. However, its dissemination becoming a more widespread practice is indeed a desired perspective not only for uterus transplantation but also for other modalities of living donor transplantation, such as kidney and liver^4,39^. 

 Another perspective is that uterus transplantation be implemented as an assistance protocol and no longer just as a research project in Brazil. In the United States, some centers already perform this transplant as care^12^, and Germany was the first country in the world where the government will cover the costs of the procedure, which will probably spread soon throughout the European Union^29^. Sweden has also recently started offering uterine transplantation as a therapeutic option for cases of absolute uterine factor infertility^4^. 

 Perhaps, in the not-too-distant future, the uterus from a matrix will replace living and deceased donors. In a recent 2023 article in Biomedical Materials and Devices, the authors highlight the good prospects of bioengineering in uterus transplantation^29^. 

## CONCLUSIONS

 Uterus transplantation is a new modality among organ transplants. It has undoubtedly brought great innovation and excellent results to the therapeutic armamentarium for absolute uterine factor infertility. Uterus transplantation is a great example where multidisciplinarity, in which each contributes with their specific knowledge, enabled the achievement of a greater feat. 

 The Department of Gastroenterology, together with the Department of Gynecology and Obstetrics, combined their knowledge and efforts and achieved great success in this new field of transplantation. There is a great expansion of uterus transplantation worldwide, now performed on all continents. The gain of experience, technology, and minimally invasive surgeries will certainly bring even better results. 

## Data Availability

The datasets generated and/or analyzed during the current study are available from the corresponding author upon reasonable request.
